# Loss of miR-200c up-regulates CYP1B1 and confers docetaxel resistance in renal cell carcinoma

**DOI:** 10.18632/oncotarget.3484

**Published:** 2015-03-08

**Authors:** Inik Chang, Yozo Mitsui, Shinichiro Fukuhara, Ankurpreet Gill, Darryn K. Wong, Soichiro Yamamura, Varahram Shahryari, Z. Laura Tabatabai, Rajvir Dahiya, Dong Min Shin, Yuichiro Tanaka

**Affiliations:** ^1^ Department of Surgery and Division of Urology, Veterans Affairs Medical Center, San Francisco, California, United States of America; ^2^ Department of Oral Biology, Yonsei University College of Dentistry, Seoul, South Korea; ^3^ Department of Urology, University of California, San Francisco, California, United States of America; ^4^ Department of Pathology, Veterans Affairs Medical Center and University of California, San Francisco, California, United States of America

**Keywords:** Renal cell carcinoma, CYP1B1, miR-200c, chemotherapy, docetaxel

## Abstract

Despite high protein expression and enzymatic activity of cytochrome P450 1B1 (CYP1B1) in renal cell cancer (RCC), its functional significance has not been elucidated. Here we explored the functional role and regulatory mechanism of CYP1B1 in RCC. Reduction of CYP1B1 levels fail to prevent *in vitro* tumorigenicity such as proliferation, apoptosis, and cell cycle progression of RCC cells. Moreover, the expression levels are not associated with tumor type, stage, Fuhrman grade and 5-year survival probability after surgery. Instead, alteration of CYP1B1 expression regulates the chemosensitivity of RCC cells to docetaxel suggesting its critical contribution to the chemoresistance. Additionally, miR-200c, which is significantly down-regulated in RCC regulates CYP1B1 expression and activity. An inverse association was also observed between the expression levels of miR-200c and CYP1B1 protein in RCC tissues. Finally, alteration of miR-200c levels affects the chemosensitivity of RCC cells. Restoration of docetaxel resistance by exogenous expression of CYP1B1 in miR-200c-over-expressing cells indicates that CYP1B1 is a functional target of miR-200c. These results suggest that CYP1B1 up-regulation mediated by low miR-200c is one of the mechanisms underlying resistance of RCC cells to docetaxel. Therefore, expression of CYP1B1 and miR-200c in RCC may be useful as a prediction for docetaxel response.

## INTRODUCTION

Renal cell cancer (RCC) accounts for 2-3% of all tumor malignancies in human. In 2008, an estimated 54,000 adults in the United States was diagnosed with RCC and approximately one third of newly diagnosed patients present with metastases [1]. The 5-year survival rate for patients with metastatic RCC is less than 10% probably due to its low response to chemo- and radiotherapy [2].

Cytochrome P450 (CYP) is a multi-gene family of enzymes implicated in the metabolism of a diverse range of xenobiotics and endogenous compounds. Cytochrome P450 1B1 (CYP1B1) belongs to the CYP1 family and is the only member of the CYP1B subfamily. It is involved in the hydroxylation of estrogen and activation of environmental carcinogens [3]. CYP1B1 expression can be induced by polycyclic aromatic hydrocarbons such as 2, 3, 7, 8-tetrachlorodibenzo-*p*-dioxin and is also regulated by peptide hormones, cAMP, aryl hydrocarbon receptor and estrogen receptor [4, 5]. CYP1B1 protein is found in cancer cells but is undetectable in the normal cells of cancerous and normal tissues [6]. In contrast, there is no obvious difference in CYP1B1 mRNA levels between cancer and normal tissues, suggesting that its expression is regulated at the post-transcriptional level. Indeed, proteasome- and microRNA (miRNA)-mediated CYP1B1 regulation have been suggested to explain the discrepancy [7, 8]. The tumorigenic effects of CYP1B1 are believed to be its ability to produce 4-hydroxy estrogens via hydroxylation of the parent estrogen [9]. 4-Hydroxy-estrogen undergoes further metabolic conversion to other reactive compounds such as estradiol-3, 4-quinone and semi-quinones. It has been postulated that DNA damage, mutation, and depurination caused by the metabolites of CYP1B1 activation initiate carcinogenesis [10]. In addition, CYP1B1 up-regulation is also thought to contribute to the chemoresistant phenotype of cancers. CYP1B1 overexpression decreased sensitivity to docetaxel *in vitro* [11] and its polymorphic allele leading both increased expression and activity is associated with poor response to taxanes in breast and prostate cancers [12, 13]. Due to these characteristics, CYP1B1 has been recognized as a potential tumor biomarker and a promising target for anticancer therapy. In fact, it has been demonstrated that CYP1B1 inhibition prevents endometrial and head and neck carcinogenesis [14, 15]. However, despite its potential tumorigenic effects, little is known about the functional significance of CYP1B1 in RCC.

MiRNAs are small noncoding RNAs of approximately 20-23 nucleotides that function in posttranscriptional gene regulatory pathways via targeting the 3′-untranslated region (UTR) of mRNAs [16]. Numerous studies have demonstrated that miRNAs have fundamental roles in many key cellular processes, such as proliferation, differentiation and apoptosis, and its altered expression has been described in many human cancers [17]. The miR-200 family containing miR-200a, -200b, -200c, -141 and -429 has been shown to play an important role in epithelial to mesenchymal transition (EMT) by repressing zinc finger E-box binding homeobox proteins 1 and 2, and inducing E-cadherin expression [18]. Loss of miR-200 family members occurs in many different human cancers and miR-200c is down-regulated in RCC [19–21]. In addition to its role in EMT, miR-200c has been shown to regulate drug resistance of various types of cancers such as bladder [22], non-small cell lung [23], esophageal [24], breast [25], and ovarian [26] cancers. Although low response to chemotherapeutics is a distinctive characteristic of RCC, it is not known whether miR-200c is involved in RCC chemoresistance.

In this study, we show that CYP1B1 up-regulation, which is associated with the reduced miR-200c expression, is involved in the chemosensitivity of RCC cells to docetaxel.

## RESULTS

### Functional role of CYP1B1 in RCC cells

**Figure 1:**
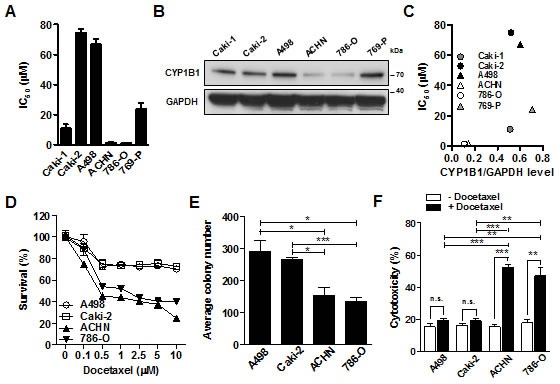
CYP1B1 expression is involved in the docetaxel resistance of RCC cells (A) Chemosensitivity assay was conducted at 72 h after docetaxel treatment. Values indicate mean IC_50_ ± SEM. (B) CYP1B1 protein expression in RCC cells was determined by Western blot. (C) The correlation of CYP1B1 protein expression with IC_50_. CYP1B1 protein level normalized by GAPDH expression was plotted against IC_50_ of different RCC cells. (D) Dose-dependent response of RCC cells was examined at 72 h after indicated concentration of docetaxel treatment. (E) Colony formation assay was performed at 14 days after docetaxel (5 μM) treatment. *P < 0.05; ***P < 0.001 (F) Cytotoxicity was measured with annexin V-FITC/7-AAD staining at 72 h after docetaxel (5 μM) treatment. *P < 0.05; **P < 0.01

To determine the functional significance of CYP1B1 in RCC, we examined whether reduction of CYP1B1 levels affects *in vitro* tumorigenicity. Transfection with two different CYP1B1 siRNAs resulted in a dramatic reduction in endogenous levels of CYP1B1 mRNA in A498 cells (Supplementary Fig. S1A). However, CYP1B1 knockdown did not affect cell proliferation, apoptosis and cell cycle regulation (Supplementary Fig. S1B–D). Since the association of CYP1B1 with the docetaxel response has been studied both *in vitro* and *in vivo* [11–13], we examined the potential function of CYP1B1 in docetaxel resistance of RCC cells. According to the cytotoxic activity of docetaxel in several RCC cell lines, the resistance was found to be dependent on the cell line. Thus, A498 and Caki-2 cells exhibited high levels of resistance with IC_50_ values of 74.6 and 66.8 μM, respectively, whereas ACHN and 786-O cells were significantly sensitive to docetaxel with IC_50_ values of 1.55 and 1.3 μM, respectively. Caki-1 and 769-P cells showed a middle range of IC_50_ values of 11.0 and 24.0 μM, respectively (Fig. 1A). Next, we examined whether CYP1B1 expression is associated with the chemosensitivity to docetaxel. As shown in Fig. 1B and C, CYP1B1 protein levels in A498 and Caki-2 cells which present a resistant phenotype were relatively higher than that of ACHN and 786-O cells which were sensitive to docetaxel. In addition, after docetaxel treatment, ACHN and 786-O cells expressing relatively low levels of CYP1B1 protein exhibit reduced survival rate and less colony-forming ability than A498 and Caki-2 cells expressing relatively high levels of CYP1B1 protein (Fig. 1D and E). Flow cytometry analysis revealed a more significant increase in cell death of ACHN and 786-O cells than A498 and Caki-2 cells (Fig. 1F). Since taxane-induced cytotoxicity is controlled by CYP2C8 [27], CYP3A4 [28], and ABCB1 [29], we examined their protein expression in RCC cells. However, expression of these proteins was not correlated with the sensitivity to taxanes (data not shown). These results suggest that CYP1B1 may be involved in the regulation of RCC cell response to docetaxel.

### CYP1B1 regulates RCC cell resistance docetaxel

**Figure 2:**
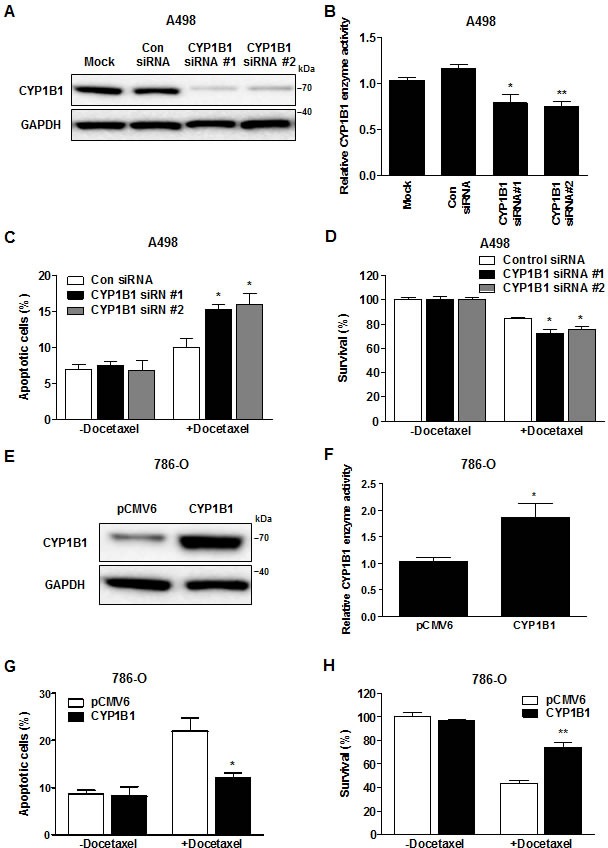
Change in CYP1B1 expression alters docetaxel resistance of RCC cells (A and B) After siRNA transfection, CYP1B1 protein expression was determined by Western blot (A) and enzyme activity was measured with P450-Glo assay (B) in A498 cells. *P < 0.05; **P < 0.01 (C and D) After CYP1B1 siRNA transfection, A498 cells were treated with docetaxel (5 μM) for 72 hrs. Apoptotic cell death was measured with annexin V-FITC/7-AAD staining (C) and cell survival was analyzed by MTS assay (D). *P < 0.05 (E and F) After plasmid transfection, CYP1B1 protein expression was determined by Western blot (E) and enzyme activity was measured with P450-Glo assay (F) in 786-O cells. *P < 0.05 (G and H) After CYP1B1 plasmid transfection, 786-O cells were treated with docetaxel (5 μM) for 72 hrs. Apoptotic cell death was measured with annexin V-FITC/7-AAD staining (G) and cell survival was analyzed by MTS assay (H). *P < 0.05; **P < 0.01.

To determine whether the regulation of CYP1B1 levels affects chemosensitivity to docetaxel, we performed knockdown experiments using CYP1B1 specific siRNA in A498 cells, which express relatively high levels of endogenous CYP1B1 protein. Transfection with two different CYP1B1 siRNAs results in a dramatic reduction in endogenous levels of CYP1B1 mRNA (Supplementary Fig. S1A), protein (Fig. 2A) and enzyme activity (Fig. 2B). CYP1B1 knockdown significantly increased the cytotoxic effect of docetaxel (Fig. 2C) and decreased the survival rate of A498 cells (Fig. 2D). In reciprocal experiments, we examined whether CYP1B1 over-expression increased the chemoresistance of RCC cells to docetaxel. Transient transfection of a plasmid containing human CYP1B1 into 786-O cells, which express relatively low levels of endogenous CYP1B1 protein resulted in a significant increase in protein level (Fig. 2E) and enzyme activity compared to transfection of control vector (Fig. 2F). Over-expression of CYP1B1 reduced the cytotoxic effect of docetaxel (Fig. 2G) and as a consequence, the survival of 786-O cells was also enhanced (Fig. 2H). Collectively, these results indicate that CYP1B1 plays an important role in the response of RCC cells to docetaxel.

### Clinicopathologic analysis of CYP1B1 expression in RCC tissues

**Figure 3:**
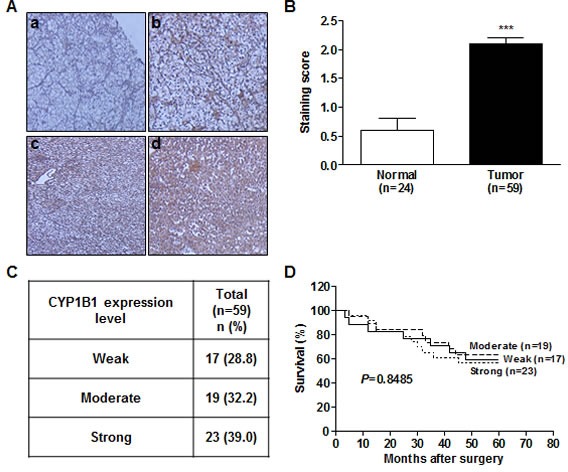
CYP1B1 protein is up-regulated in RCC tissues (A) Immunohistochemical staining of CYP1B1 protein expression in RCC patients tissues. (a-d) Representative images showing immunoreactive CYP1B1 in normal (a) and RCC (b-d) tissues (magnification: ×400). (B) Summary of CYP1B1 immunostaining score. Staining intensity was assessed as described in *Materials and Methods*. ***P < 0.001 (C) Summary of CYP1B1 expression levels in RCC tissue samples. (D) Kaplan-Meier analysis for the correlation of CYP1B1 expression with 5 year survival after surgery in RCC patients.

Despite high protein expression and elevated enzymatic activity of CYP1B1 in RCC [6, 30], the association of CYP1B1 with RCC development and clinicopathologic relevance is unknown. To determine whether the CYP1B1 protein expression levels are related with the clinicopathologic features and risk of RCC progression, we analyzed 59 patient samples who underwent nephrectomy for RCC, 24 of whom also had adjacent normal kidney samples available for analysis by immunohistochemistry. While the CYP1B1 level was weak or not detected in most of the normal tissues, majority of RCC tissue samples showed moderate or strong CYP1B1 immunoreactivity with average staining scores of 2.10 ± 0.11 (versus 0.60 ± 0.20 in normal tissues) (Fig. 3A and B). Unlike protein expression, no significant difference was observed in the levels of CYP1B1 mRNA in both RCC tissues and cell lines (Supplementary Fig. S2). According to the scoring, we categorized the RCC samples into weak (28.8%), moderate (32.2%) and strong (39.0%) staining groups (Fig. 3C). As shown in Fig. 3D, no correlation was observed between CYP1B1 levels and 5-year survival probability after surgery (*P* = 0.8485). Of the RCC tissue samples, 49 of 59 (83%) were clear cell and 7 of 59 (11.9%) were papillary subtypes. In addition, 3 of 59 (5.1%) tumors were identified as granular, tubular carcinoma, or oncocytoma (Table 1). We further assessed whether CYP1B1 expression in clinical samples are correlated with clinicopathologic characteristics such as tumor types, stage and Fuhrman grade. However, no statistically significant correlation was observed between CYP1B1 expression and these pathological parameters. A comprehensive summary of the CYP1B1 staining patterns and its association with several clinicopathologic variables is shown in Table 1.

**Table 1:** Association of CYP1B1 expression with the clinicopathologic characteristics of RCC

Pathologicalvariables	Total (*n* = 59),n (%)	CYP1B1 expression			*P*
		Weak (*n* = 17)	Moderate (*n* = 19)	Strong (*n* = 23)	
**Age**					
Mean ± SD, years	66.2 ± 6.8	-	-	-	
**Sex**					
Men	37/59 (62.7)	12/37 (45.9)	10/37 (27.0)	15/37 (40.5)	0.5201
Women	22/59 (37.2)	5/22 (22.7)	9/22 (40.9)	8/22 (36.4)	
**Histological type**					
Clear cell	49/59 (83.0)	13/49 (26.5)	17/49 (34.7)	19/49 (38.8)	0.5204
Papillary	7/59 (11.9)	2/7 (28.6)	1/7 (14.3)	4/7 (57.1)	
Granular	1/59 (1.7)	-	1/1 (100)	-	
Tubular	1/59 (1.7)	1/1 (100)	-	-	
Oncocytoma	1/59 (1.7)	1/1 (100)	-	-	
**Tumor stage**					
pT1	31/59 (52.5)	9/31 (29.0)	7/31 (22.6)	11/31 (35.5)	0.0977
pT2	11/59 (18.6)	2/11 (18.2)	4/31 (12.9)	9 (81.8)	
pT3	13/59 (22.0)	6/13 (46.2)	6/13 (46.2)	1/13 (7.7)	
pT4	4/59 (6.8)	-	2/4 (50.0)	2/4 (50.0)	
**Nuclear grade**					
1	35/59 (59.3)	14/35 (40.0)	12/35 (34.3)	9/35 (25.7)	0.1656
2	14/59 (23.7)	2/14 (14.3)	3/14 (21.4)	9/14 (64.2)	
3	6/59 (10.2)	1/6 (16.7)	2/6 (33.3)	3/6 (50.0)	
4	4/59 (8.5)	-	2/4 (50.0)	2/4 (50.0)	
**Vascular invasion**					
Negative	56/59 (94.9)	-	-	-	
Positive	3/59 (5.1)	-	-	-	
**Capsular invasion**					
Negative	57/59 (96.6)	-	-	-	
Positive	2/59 (3.4)	-	-	-	

### CYP1B1 level is inversely correlated with miR-200c expression in RCC

**Figure 4:**
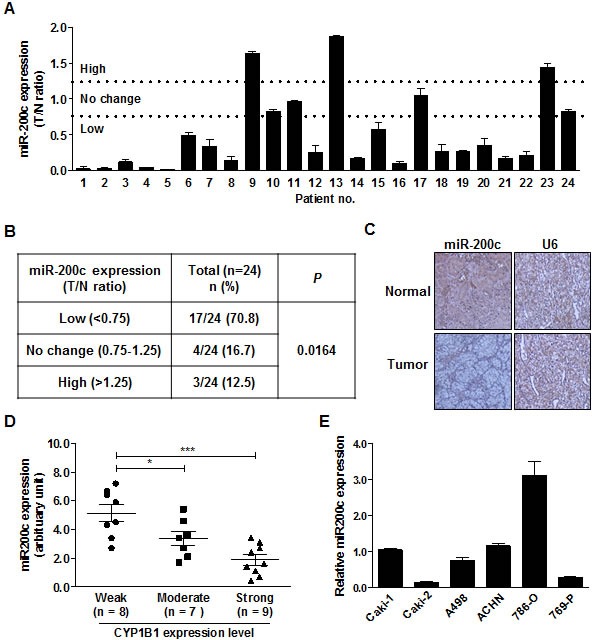
Inverse correlation between endogenous miR-200c level and CYP1B1 protein expression (A) MiR-200c expression in RCC tissues. MiR-200c levels were analyzed by RT-PCR with RNA from microdissected tumor (T) and adjacent normal (N) tissues. (B) Summary of miR-200c expression levels in RCC tissue samples. (C) *In situ* hybridization of miR-200c expression in normal and RCC tissues. (D) Association of CYP1B1 expression with miR-200c levels in RCC tissues. MiR-200c levels were measured by RT-PCR with RNA extracted from different scored and microdissected RCC tissue samples. *P < 0.05; ***P < 0.001 (E) Relative miR-200c expression in RCC cells was analyzed by RT-PCR.

Since CYP1B1 overexpression causes resistance of RCC cells to docetaxel, we looked of the regulatory mechanisms of CYP1B1 expression with a focus on miRNAs. We searched for potential candidate miRNAs, which have been reported to be altered in RCC. According to miRNA expression profiling studies, miR-200c is significantly down-regulated in RCC [19, 20]. Therefore, we analyzed miR-200c expression in microdissected human renal cancer tissues and matched adjacent normal regions, which were used for the CYP1B1 expression study (n = 24). While the expression of miR-200c was unaltered in 4 samples or up-regulated in 3 of the 24 cases, most of RCC tissues (17 of 24 cases) had low miR-200c levels relative to the matched normal samples (Fig. 4A and B). We verified these results by *in situ* hybridization analysis and again observed attenuated miR-200c expression (Fig. 4C). Patient and tumor characteristics used in these studies are summarized in Supplementary Table S1.

To determine whether miR-200c expression is associated with CYP1B1 level, we investigated the relationship between the levels of CYP1B1 protein and miR-200c in human RCC tissues (Fig. 4D). RCC tumors with strong CYP1B1 expression showed low miR-200c levels (1.87 ± 0.36 versus 5.14 ± 0.58 in tumor samples with weak CYP1B1 expression). In addition, a significant difference was also observed between RCC tumors with moderate and low CYP1B1 levels (3.36±0.50 versus 5.14±0.58 in tumor samples with weak CYP1B1 expression). Thus, an inverse association was found between miR-200c expression and CYP1B1 protein levels in human RCC tissues. In addition to the majority of RCC cell lines showed low expression of miR-200c compared with HK-2 cells but 786-O cells, which express low CYP1B1 levels exhibited high miR-200c expression (Fig. 4E). In contrast to the CYP1B1 protein level, no relationship was found between CYP1B1 mRNA and miR-200c expression in the human RCC tissues (Supplementary Fig. S3). These results suggest that low expression of miR-200c results in high levels of CYP1B1 protein in RCC tumors.

### CYP1B1 is a direct target of miR-200c

**Figure 5:**
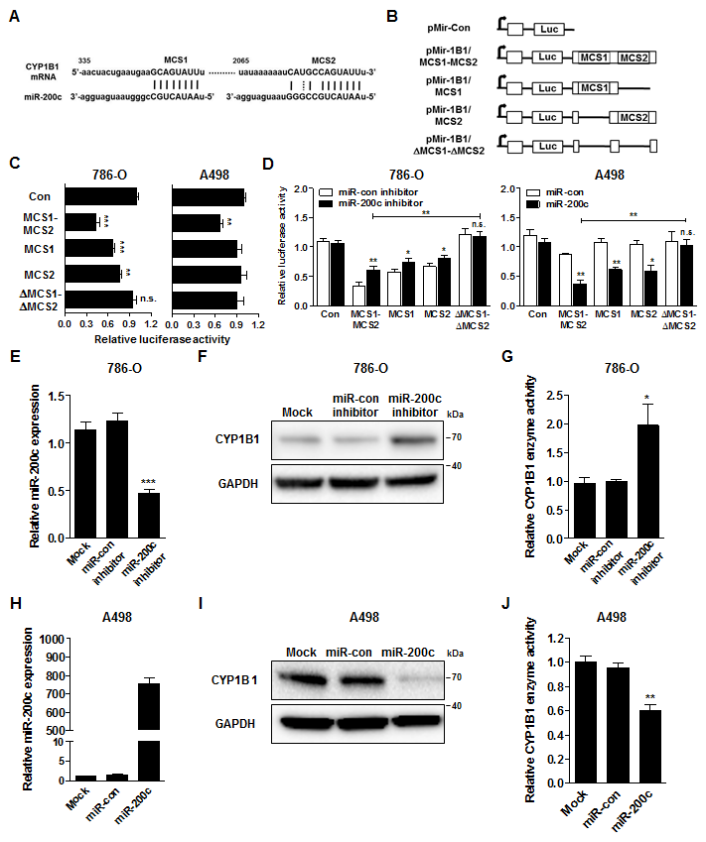
MiR-200c directly targets the CYP1B1 3′-UTR (A) Sequence and location of MCS1 and 2 in the 3′-UTR of human CYP1B1 mRNA. (B) Diagram of reporter constructs containing the wild type or MCS-deleted 3′-UTR of human CYP1B1. (C) Relative luciferase activity after transfection of reporter constructs containing the wild type or MCS-deleted 3′-UTR of CYP1B1. **P < 0.01; ***P < 0.001; n.s.: non-significant (D) Relative luciferase activity after co-transfection of reporter constructs containing the wild type or MCS-deleted 3′-UTR of CYP1B1with either miR-200c inhibitor or precursor. *P < 0.05; **P < 0.01; n.s.: non-significant (E-G) After miR-200c inhibitor transfection, relative miR-200c expression was analyzed by RT-PCR (E), CYP1B1 protein level was determined by Western blot (F) and enzyme activity was measured with P450-Glo assay (G) in 786-O cells. *P < 0.05; ***P < 0.001 (H-J) After miR-200c precursor transfection, relative miR-200c expression was analyzed by RT-PCR (H), CYP1B1 protein level was determined by Western blot (I) and enzyme activity was measured with P450-Glo assay (J) in A498 cells. **P < 0.01.

*In silico* analysis (http://www.microrna.org/ and http://targetscan.org/) showed that CYP1B1 3′-UTR contains two potential complementary miR-200c binding sequences which are termed miR-200c complementary sequence 1 and 2, respectively (MCS1 and MCS2) (Fig. 5A). To examine whether CYP1B1 expression is regulated by miR-200c, we cloned various 3′UTR reporter constructs containing the MCSs or controls (Fig. 5B) and conducted luciferase assays with 786-O and A498 cells, respectively. With 786-O cells, the luciferase activity of the construct containing two copies (pMir-1B1/MCS1-MCS2) or one copy (pMir-1B1/MCS1 or pMir-1B1-MCS2) of MCS was significantly lower than that of the control plasmid. However, the luciferase activity of the construct containing no MCS (pMir-1B1/ΔMCS1-ΔMCS2) showed no change compared to the control vector. In A498 cells, only the luciferase activity of pMir-1B1/MCS1-MCS2 was significantly decreased probably due to the low endogenous miR-200c expression. To investigate the direct regulation of CYP1B1 expression by miR-200c, luciferase activities were measured after miR-200c inhibition or overexpression. As shown in Fig. 5D, inhibition of endogenous miR-200c increased the luciferase activities of the pMir-1B1/MCS1-MCS2 but not that of pMir-1B1/ΔMCS1-ΔMCS2. In reciprocal experiments, exogenous miR-200c expression decreased the luciferase activities of the pMir-1B1/MCS1-MCS2 but not that of pMir-1B1/ΔMCS1-ΔMCS2. These results suggest that miR-200c directly targets the MCS on the CYP1B1 3′-UTR and regulates its expression.

Next, we examined the effect of miR-200c on CYP1B1 protein levels and its enzymatic activity. In 786-O cells, endogenous miR-200c levels were decreased by the miR-200c inhibitor (Fig. 5E). As shown in Fig. 5F and G, the CYP1B1 protein level and enzyme activity were significantly increased by the miR-200c inhibition. In reciprocal experiments, we also looked the effects of miR-200c overexpression in A498 cells. MiR-200c expression was greatly increased by the transfection of its precursor in A498 cells (Fig. 5H). Conversely, CYP1B1 protein and enzyme activity were significantly decreased by miR-200c overexpression (Fig. 5I and J). These results suggest that miR-200c represses CYP1B1 mRNA expression resulting in the reduction of CYP1B1 protein level and enzymatic activity.

### MiR-200c-mediated CYP1B1 regulation affects the resistance of RCC cells to docetaxel

**Figure 6:**
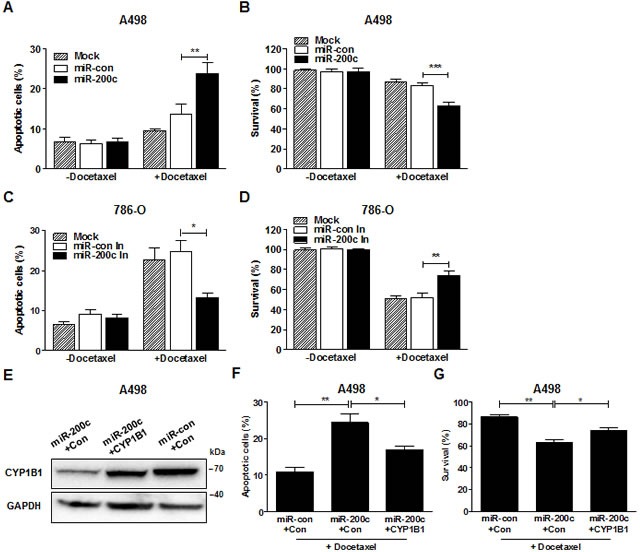
MiR-200c regulates docetaxel resistance of RCC cells (A and B) After miR-200c precursor transfection, A498 cells were treated with docetaxel (5 μM) for 72 hrs. Apoptotic cell death was measured with annexin V-FITC/7-AAD staining (A) and cell survival was analyzed by MTS assay (B). **P < 0.01; ***P < 0.001 (C and D) After miR-200c inhibitor transfection, 786-O cells were treated with docetaxel (5 μM) for 72 hrs. Apoptotic cell death was measured with annexin V-FITC/7-AAD staining (C) and cell survival was analyzed by MTS assay (D). *P < 0.05; **P < 0.01 (E-G) After transfection of miR-200c precursor with either control or CYP1B1 plasmid, A498 cells were treated with docetaxel (5 μM) for 72 hrs. CYP1B1 protein expression was determined by Western blot (E), apoptotic cell death was measured with annexin V-FITC/7-AAD staining (F) and cell survival was analyzed by MTS assay (G). *P < 0.05; **P < 0.01.

To determine whether miR-200c affects RCC chemosensitivity, the cytotoxic effect of docetaxel was measured after miR-200c restoration or inhibition. A significant increase in the cytotoxic effect of docetaxel was observed and as a consequence, the cell survival rate was reduced by transfection of miR-200c precursor in A498 cells (Fig. 6A and B). MiR-200c inhibition significantly decreased the cytotoxic effect of docetaxel and enhanced the survival rate of 786-O cells (Fig. 6C and D). To verify the effects of miR-200c via CYP1B1 on the chemosensitivity to docetaxel, we introduced CYP1B1 plasmid into A498 cells after miR-200c overexpression and examined cytotoxicity. Restoration of CYP1B1 protein levels was verified after transfection (Fig. 6E). We observed that the increase in the cytotoxic effect of docetaxel by miR-200c overexpression was reversed by the enforced CYP1B1 expression (Fig. 6F). As a consequence, cell survival was also restored by the exogenous CYP1B1expression (Fig. 6G). These results indicate that CYP1B1 regulation by miR-200c directly influences the chemosensitivity of RCC cells to docetaxel.

## DISCUSSION

It has been suggested that CYP1B1 may be important in tumor development and progression, and also contributes to drug resistance [31, 32]. Saini *et al.* showed that CYP1B1 up-regulation plays an essential role in endometrial carcinogenesis [14]. In head and neck carcinogenesis, CYP1B1 knockdown reduced the migration and proliferation of premalignant cells [15]. Based on these studies, we examined the functional role of CYP1B1 in RCC using siRNA-mediated CYP1B1 knockdown. Unlike other studies, CYP1B1 deficiency had no influence on RCC tumorigenicity. Interestingly, we found that CYP1B1 is involved in the docetaxel resistance of RCC cells. CYP1B1 overexpression in V79 cells reduced the *in vitro* cytotoxicity of docetaxel and this effect was reversed in the presence of CYP1B1 inhibitor [11]. Although it is controversial [33, 34], one of the CYP1B1 polymorphic variants, Leu432Val, which may increase enzymatic activity, is associated with low response to taxane in breast cancer [12] and poor prognosis in docetaxel-treated prostate cancer patients [13, 35]. The L432V allelic variants were also associated with reduced sensitivity to DNA-interacting agents, alkylators, camptothecins, topoisomerase II inhibitors, and antimetabolites [36]. Our previous study suggests that CYP1B1 gene polymorphisms are risk factors for RCC [37]. Therefore, it would be of interest to determine whether the CYP1B1 polymorphic variants, especially the L432V allelic variant, are related to the resistance of RCC to chemotherapeutics.

Several hypotheses have been proposed to explain how CYP1B1 decreases the cytotoxic effect of docetaxel. Rochat *et al.* initially suggested that docetaxel is metabolized by CYP1B1 [32]. However, other studies were unable to detect catalytic activity and effect of CYP1B1 during the metabolism and clearance of docetaxel, respectively [13, 38]. Sissung *et al.* showed that both 2-OHE_2_ and 4-OHE_2_, the catechol estrogen metabolites of CYP1B1 hamper the docetaxel-mediated tubulin polymerization. They also presumed that the covalent binding of CYP1B1-catalyzed E2-3, 4-quinone to docetaxel may reduce the cytotoxic potency of the drug [13]. CYP1B1 expression or its metabolites may also trigger an intracellular protective signaling pathway against docetaxel cytotoxicity. Martinez *et al.* showed that docetaxel induces CYP1B1 expression and drug resistance in breast but not in lung, colon, or pancreatic cancer cells. They also suggested that docetaxel resistance is not due to the direct inactivation of docetaxel by CYP1B1 but rather the indirect mechanism of promoting cancer cell survival based on the pro-apoptotic effect of CYP1B1 knockdown in endometrial cancer cells [39]. However, in RCC cells, CYP1B1 reduction does not affect tumorigenicity and no docetaxel-mediated CYP1B1induction was observed (Supplementary Fig. S1 and S4). Therefore, it may be possible that CYP1B1 is involved in docetaxel resistance through diverse mechanisms in each different type of cancer. MiR-200c is an important regulator of EMT, which has been suggested as one of the regulatory mechanisms of drug resistance [40]. According to our study, CYP1B1 is an essential target of miR-200c-mediated docetaxel resistance. Thus, further studies are needed to determine whether CYP1B1 is involved in EMT.

CYP1B1 has been considered to be a promising prognostic and diagnostic marker for RCC, however no studies have been conducted to document it so far. In this study, we examined the association between CYP1B1 expression and RCC clinicopathologic factors. However, no correlation was observed between CYP1B1 expression and RCC tumor type, stage, grade, and 5-year survival probability after surgery. Importantly, we observed that CYP1B1 expression is associated with the chemosensitivity of RCC cells to docetaxel. Although our sample number is small, it was enough to obtain statistical significance. A larger sample size might be needed to draw a more concrete conclusion about the association between CYP1B1 expression and clinical indicators of RCCs. In addition, it will be helpful to perform studies on the association of CYP1B1 expression and its polymorphic variants with the efficiency of docetaxel treatment in RCC patients to confirm this study.

Several reports have indicated that miR-200c is down-regulated in RCC tissues [19–21]. However, little is known about the functional significance of low miR-200c expression in RCC. Reduced miR-200c level is associated with aggressiveness and chemoresistance in female reproductive cancers and its restoration enhances sensitivity to paclitaxel by targeting class III beta tubulin [41, 42]. In addition, miR-200c is associated with cisplatin and doxorubicin sensitivity through regulation of Akt, AP-2α, TrkB and Bmi1 [24, 25, 43]. In this study, we identified CYP1B1 to be an effector for miR-200c-mediated regulation of RCC chemosensitivity to docetaxel. Therefore, it would be of interest to examine whether genes known to be related with drug resistance such as ATP-binding cassette transporters and major vault protein are also regulated by miR-200c. According to Tsuchiya *et al*., lack of miR-27b which inhibits the translation of CYP1B1 mRNA into protein in normal but not cancer cells causes up-regulation of CYP1B1 protein level in breast cancer cells [7]. Therefore, it would be of great interest to determine whether miR-27b-mediated CYP1B1 regulation is involved in docetaxel resistance in both breast and kidney cancers. The information obtained by these types of studies will provide additional insight into understanding the significance of miRNAs in RCC drug resistance.

In conclusion, the present findings indicate that CYP1B1 is post-transcriptionally regulated by miR-200c and thus, high CYP1B1 protein expression and enzyme activity may be caused by low expression of miR-200c in RCC. CYP1B1 up-regulation is not related to tumorigenicity and is unlikely to be associated with RCC clinicopathological parameters. Interestingly, our study found that miR-200c-mediated CYP1B1 regulation is involved in the chemosensitiviy of RCC to docetaxel. These results indicate that CYP1B1 and miR-200c may be useful as potential biomarkers for chemotherapy and therefore, help to provide more options for RCC treatment.

## MATERIALS AND METHODS

### Cell lines and reagents

The renal cancer cell lines, Caki-1, Caki-2, A498, ACHN, 786-O and 769-P were obtained from the American Type Culture Collection. McCoy's 5A, MEM Eagle's with Earle's BSS (EMEM), RPMI 1640, Opti-MEM and penicillin/streptomycin mixtures were obtained from the UCSF Cell Culture Facility. Fetal bovine serum (FBS) was a product of Atlanta Biologicals. Docetaxel was purchased from Sigma Chemical Co.

### Cell culture

Caki-1 and Caki-2 cells were grown in McCoy's 5A, and A498 and ACHN cells were maintained in EMEM medium while 786-O and 769-P cells were cultured in RPMI 1640 medium. All culture medium contained 10% FBS and 100 μg/ml penicillin/streptomycin. All cell lines were maintained at 37°C in a humidified atmosphere composed of 5 % CO_2_ /95 % air.

### Cell viability assay

For chemosensitivity assay, cells were seeded in triplicate in 96-well plates at a density of 250-1,000 cells/well as appropriate and grown for 24h and then treated with docetaxel at 10 serially diluted concentrations between 0.05 and 100 μM for 72 h. For cell survival assay, cells were plated in triplicate in 96-well plates at a density of 5,000 cells/well and then treated with docetaxel. In some cases, miR-200c precursor, inhibitor, CYP1B1 plasmid or siRNAs along with their controls were transfected before treatment. At the desired time point, the number of viable cells was determined by adding 3-(4,5-dimethylthiazol-2-yl)-5-(3-carboxymethoxyphenyl)-2-(4-sulfophenyl)-2H-tetrazolium (MTS)-based CellTiter 96 AQ_ueous_ One Solution Reagent (Promega) to each well and measuring the absorbance at 490 nm on a SPECTRA MAX 190 plate reader (Molecular Devices). Results were expressed as percentage of optical density, assuming that the absorbance of control cells was 100%.

### Western blot analysis

Whole cell extracts were prepared using radioimmunoprecipitation assay buffer (Thermo Scientific) containing protease inhibitor cocktails (Roche). Antibodies against CYP1B1 (Abcam) and glyceraldehyde-3-phosphate dehydrogenase (GAPDH; Santa Cruz Biotechnology) were used to detect protein expression.

### Colony formation assay

Cells were seeded at 1,000 cells/plate and treated with doxetacel. After 14 days incubation, the colony were fixed with glutaraldehyde (6.0% v/v), stained with crystal violet (0.5% w/v) and counted with a microscope.

### Apoptosis assay

Apoptosis was analyzed with an annexin V-fluorescein isothiocyanate (FITC)/7-amino-actinomycin D (7-AAD) staining system (BD Biosciences) and a Cell Lab Quanta™ SC MPL (Beckman Coulter). Cells were stained with annexin V-FITC only (early apoptotic) or both annexin V-FITC and 7-AAD (late apoptotic) were considered as the apoptotic cell fractions.

### CYP1B1 enzymatic activity

CYP1B1 enzymatic activity was determined using a P450-Glo Assay kit (Promega) according to the manufacturer's instructions. The enzymatic activity was normalized to the protein content.

### Transfection

For CYP1B1 overexpression, cells were transfected with pCMV6-ENTRY vector expressing human CYP1B1 cDNA or empty pCMV6-ENTRY vector (OriGene Technologies) using Fugene HD Transfection Reagent (Roche Diagnostics) according to the manufacturer's protocol. For CYP1B1 knockdown, cells were transfected with 10 nM CYP1B1 siRNAs or universal scrambled negative control (OriGene Technologies). For miR-200c inhibition, cells were transfected with 20 nM Anti-miR miRNA Inhibitor for has-miR-200c or Anti-miR miRNA Inhibitor Negative Control (Ambion). For miR-200c overexpression, cells were transfected with 50 nM Pre-miR miRNA Precursor of has-miR-200c or Pre-miR miRNA Negative Control (Ambion). Lipofectamine^TM^ 2000 Tansfection Reagent (Invitrogen) was used for the transfection of CYP1B1 siRNA, miRNA precursor and inhibitor as described by the manufacturer's instructions. Cells treated with Lipofectamine^TM^ 2000 Transfection Reagent alone were included as a mock control for each experiment.

### Quantitative RT-PCR

Total RNA was extracted from microdissected formalin-fixed, paraffin-embedded (FFPE) tissues and cultured cells using miRNeasy Mini Kit or RNeasy Mini Kit (Qiagen) and was converted into cDNA by using TaqMan MicroRNA Reverse Transcription Kit (Applied Biosystems) or iScript^™^ cDNA Synthesis Kit (BIO-RAD) according to the manufacturer's instruction. To assess gene expression, cDNAs were amplified with the TaqMan® Gene Expression Assays and TaqMan® Fast Universal PCR Master Mix using the 7500 Fast Real-Time PCR System. The target genes and their Assay ID were as follows: CYP1B1 (Hs0016383_m1), GAPDH (Hs03929097_g1), miR-200c (002300) and RNU48 (001006). The relative level was calculated by the comparative *C*_t_ method (ΔΔ*C*_t_) using the 7500 Fast System Sequence Detection Software (Applied Biosystems).

### RCC tissue samples

Tissue samples of radical nephrectomy were obtained from the patients enrolled in the San Francisco Veterans Affairs Medical Center (SFVAMC). Informed consent was obtained from all patients. All slides were reviewed by a board-certified pathologist for the identification of tumor foci and normal adjacent tissue. For microdissections, 10 μm-unstained sections were microdissected with an Autopix Laser Capture Microdissection System (Molecular Devices) using 4 μm sections stained with hematoxylin and eosin and marked tumor and normal adjacent areas as a template.

### Reporter assay

CYP1B1 3′UTR luciferase reporter clone was obtained from Origene Technologies along with control construct (pMirTarget). For miR-200c complementary sequence (MCS) mutants, deletion mutagenesis was conducted by overlap extension PCR [44]. The primer sequences are as follows: F1, 5′-AAAGAATTCACAAGCCAAGGAAACTTGCCA-3′; F2, 5′-CTGAATGAATTGGTAACCAGGCCATTT-3′; F3, 5′-AAAAATCATTTTAAAGGCATTAGAGTC-3′; R1, 5′-GGTTACCAATTCATTCAGTAGTTTGGT-3′; R2, 5′-GCCTTTAAAATGATTTTTTATATTCAA-3′; R3, 5′-AAAGCGGCCGCTTGGTAATGGTGTC CCAGTAT-3′. After transfection, luciferase activity was measured with a PerkinElmer 2030 Multilabel reader (PerkinElmer) using the Dual-Glo Luciferase Assay System (Promega).

### Immunohistochemistry

Immunostaining was performed on FFPE RCC sections using the VECTASTAIN ABC Kit (Vector Laboratories) according to the manufacturer's instructions. After incubation with anti-CYP1B1 antibody (Abcam), ImmPACT DAB (Vector Laboratories) was added as chromogen followed by counterstaining with hematoxylin. Staining intensity of each tissue section was visually evaluated with an Olympus BX60 microscope equipped with Spot Advanced software (Diagnostic Instruments) and was ranked on an overall scale from 0 to 3; with 0 indicating the absence of staining; 1, weak staining; 2, moderate staining; and 3, strong staining.

### *In situ* hybridization

*In situ* hybridization (ISH) was performed on FFPE RCC sections using the digoxigenin (DIG)-labeled miRCURY Locked Nucleic Acid (LNA) microRNA Detection probes for miR-200c and U6 (Exiqon) following the manufacturer's protocol. Hybridization was performed overnight at 57 °C and detected with horseradish peroxidase-conjugated anti-DIG antibody (Abcam). Abundance of target RNA was detected using ImmPACT DAB peroxidase substrate (Vector Laboratories).

### Statistical analysis

Values are presented as the mean ± standard error of mean (SEM) based on results obtained from at least three independent experiments. All statistical analyses were carried out using GraphPad PRISM Software. Two-tailed unpaired Student's *t*-test was used for comparisons between two groups. Chi-square test was used for analyzing the correlation between clinicopathologic parameters and CYP1B1 protein expression. Significance of percent survival after surgery was done with log-rank test. The difference of miR-200c expression was determined by Mann-Whitney *U* test. The relationship between the levels of CYP1B1 protein and miR-200c was investigated by ANOVA and Tukey method test. A *P* value of <0.05 was regarded as statistically significant.

## SUPPLEMENTARY MATERIAL


