# Supplementary material for: Loss of miR-200c up-regulates CYP1B1 and confers docetaxel resistance in renal cell carcinoma
Source: Oncotarget. 2015 Mar 8;6(10):7774–87. doi: 10.18632/oncotarget.3484 (PMC4480715; doi:10.18632/oncotarget.3484)
Supplement: Supplementary file 1 [file oncotarget-06-7774-s001.pdf]

Loss of miR-200c up-regulates CYP1B1 and confers docetaxel resistance in renal cell carcinoma

Supplementary Material

MATERIAL AND METHODS

Cell cycle analysis

After A498 cells were stained with 4’, 6-diamidino-2-phenylindole (DAPI), cell cycle was analyzed with a Cell Lab Quanta™ SC MPL (Beckman Coulter).

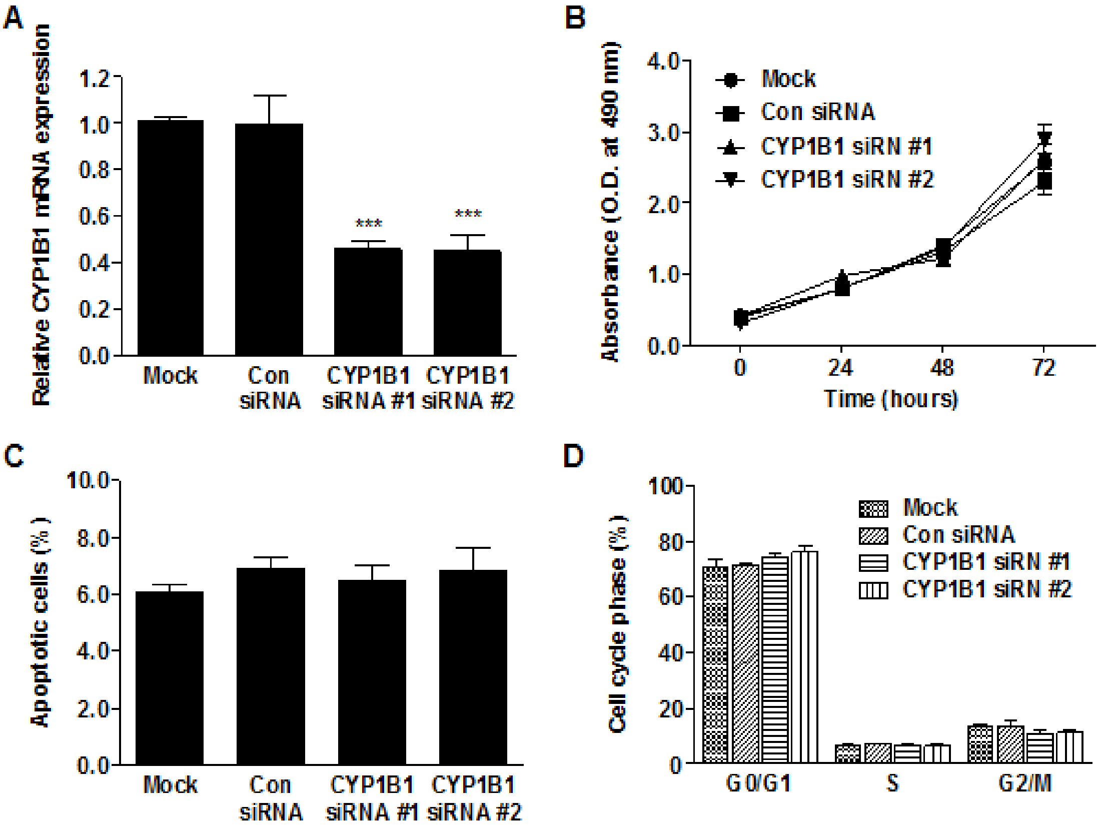

Figure S1: No effect of CYP1B1 reduction on A498 cell survival, apoptosis and cell cycle.

(A) CYP1B1 mRNA levels were determined by RT-PCR after CYP1B1 siRNA transfection. ****P*<0.001

(B-D) Cell survival was analyzed by MTS assay (B), apoptotic cell death was measured with annexin V-FITC/7-AAD staining (C) and cell cycle progression was determined by DAPI staining (D) in A498 cells after CYP1B1 siRNA transfection.

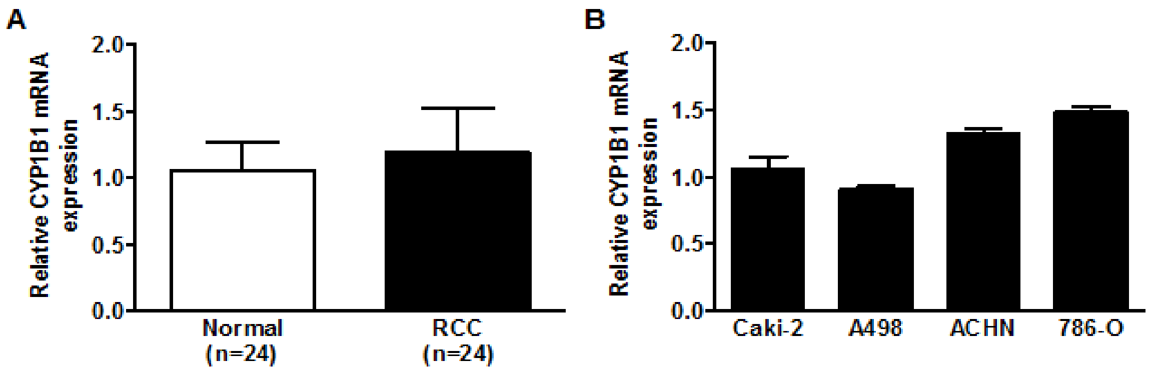

Figure S2: CYP1B1 mRNA expression in RCC tissues (A) and cell lines (B).

(A and B) CYP1B1 mRNA expression was determined by RT-PCR with RNA extracted from microdissected RCC tissues (A) and cells (B).

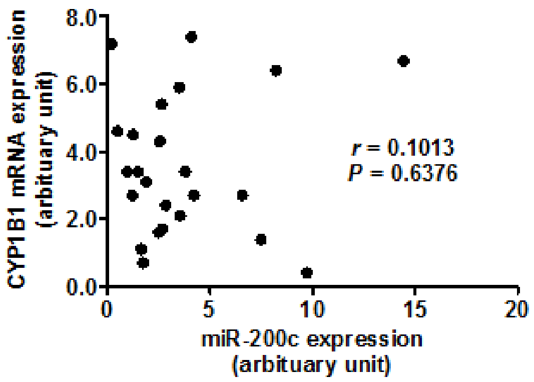

Figure S3: No correlation between CYP1B1 mRNA levels and miR-200c expression in RCC tissues.

Levels of CYP1B1 mRNA and miR-200c were determined by RT-PCR with RNA extracted from microdissected tissue samples. The relationship was analyzed with Pearson's correlation test.

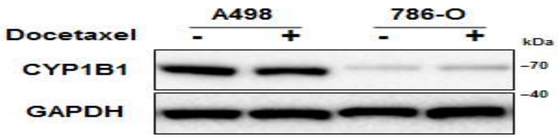

Figure S4: No induction of CYP1B1 by docetaxel in RCC cells.

CYP1B1 protein levels were determined by Western blot with total proteins harvested from cells treated with either vehicle or docetaxel (5 μM).

Table S1: Patient and tumor characteristics from clinical samples used for miR-200c expression analysis

Pathological variables	Total (n=24), n (%)
**Age**	
Mean ± SD, years	65.8 ± 7.4
**Sex**	
Men	17/24 (70.8)
Women	7/24 (29.2)
**Histological type**	
Clear cell	20/24 (83.3)
Papillary	4/24 (16.7)
**Tumor stage**	
pT1	10/24 (41.7)
pT2	6/24 (25.0)
pT3	3/24 (12.5)
pT4	5/24 (20.8)
**Nuclear grade**	
1	13/24 (54.2)
2	7/24 (29.2)
3	4/24 (16.7)
4	0/24 (0)
**Vascular invasion**	
Negative	24/24 (100)
Positive	0/24 (0)
**Capsular invasion**	
Negative	24/24 (100)
Positive	0/24 (0)
